# Phlebotomine sand fly survey, blood meal source identification, and description of *Sergentomyia imihra* n. sp. in the central Sahara of Algeria

**DOI:** 10.1186/s13071-024-06542-9

**Published:** 2024-11-04

**Authors:** Kamal Eddine Benallal, Mohammed Mefissel, Yassine Dib, Jérôme Depaquit, Daniel Kavan, Zoubir Harrat, Vít Dvořák, Petr Volf, Petr Halada

**Affiliations:** 1 Department of Parasitology, Faculty of Science, Charles University, Prague, Czech Republic; 2 Laboratory of Arboviruses and Emergent Viruses, Institut Pasteur of Algeria, Algiers, Algeria; 3 Public Establishment of Nearby Health of Illizi, Ibn-Sina, Algeria; 4 UR ESCAPE, Université de Reims Champagne-Ardenne, USC ANSES PETARD, Reims, France; 5 laboratoire de Parasitologie, Pôle de Biologie Territoriale, CHU, Reims, France; 6 BioCeV, Institute of Microbiology of The Czech Academy of Sciences, Vestec, Czech Republic; 7 Algerian Academy of Science and Technology, El Madania, Algiers Algeria

**Keywords:** *Leishmaniasis*, *Phlebotomus*, *Sergentomyia*, Barcode, Blood meal, MALDI-TOF mass spectrometry, Algeria

## Abstract

**Background:**

Phlebotomine sand flies (Diptera: Psychodidae) are important vectors of various pathogens, mainly *Leishmania* parasites. In the Old World, the most important genus in term of pathogens transmission is the genus *Phlebotomus*, which includes many proven or suspected vectors of several *Leishmania* species, while the genus *Sergentomyia* remains so far unproven as a vector of human pathogens. Algeria is one of the most affected countries by human leishmaniasis.

**Methods:**

In the present study, an entomological survey was carried out in two provinces, Ghardaïa and Illizi, located in the north and central Sahara, respectively, where cases of human leishmaniasis are recorded. Our goal was to understand the role of the local sand fly species in the transmission of *Leishmania* parasites and to analyze their blood meal preferences. Collected sand flies were identified by a combination of morphological and molecular approaches that included DNA-barcoding and matrix-assisted laser desorption/ionization time-of-flight mass spectrometry (MALDI-TOF MS) protein profiling. In addition, female blood meals were analyzed by peptide mass mapping using MALDI-TOF MS.

**Results:**

In total, 640 sand fly specimens belonging to *Phlebotomus* and *Sergentomyia* genera were collected in the two provinces. *Sergentomyia antennata* and *Se. fallax* were most abundant species in Ghardaïa, and *Ph. papatasi* and *Ph. alexandri* in Illizi. In addition, a new sand fly species was described in Illizi named *Sergentomyia* (*Sergentomyia*) *imihra* n. sp. Blood meal analysis of the engorged females revealed various mammalian hosts, especially goats, but also humans for *Phlebotomus papatasi* and *Ph. alexandri*, suggesting that these vector species are opportunistic feeders.

**Conclusions:**

Integrative approach that combined morphological analysis, sequencing of DNA markers, and protein profiling enabled the recognition and description of a new *Sergentomyia* species, raising the number of the Algerian sand fly fauna to 27 species. Further sand fly surveillance in the central Sahara is recommended to identify the thus-far unknown males of *Se. imihra* n. sp.

**Graphical Abstract:**

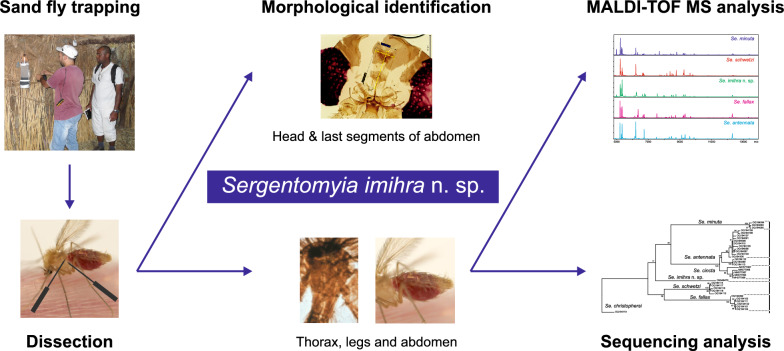

**Supplementary Information:**

The online version contains supplementary material available at 10.1186/s13071-024-06542-9.

## Background

Despite affecting human populations in 200 countries and territories worldwide, leishmaniases are still regarded as neglected tropical diseases. In 2020, more than 200,000 cases of cutaneous leishmaniasis (CL) and 12,000 cases of visceral leishmaniasis (VL) were reported. The Eastern Mediterranean region and Algeria constitute an eco-epidemiological “hotspot”, as together they contribute 79% (162 371) of all reported CL cases [1]. In Algeria, both CL and VL are endemic [2]. Cutaneous leishmaniasis occurs in almost all provinces except those of the central Sahara [3–5]. Visceral leishmaniasis occurs mainly in northern Algeria, but recently reemerging foci in the central Sahara reported the occurrence of VL cases, with an incidence higher than the historical focus in the province of Tizi Ouzou located in the northern part of the country [6–8].

Sand fly fauna of Algeria is composed by 26 species belonging to two genera, *Phlebotomus* and *Sergentomyia* [9]. Local members of the genus *Phlebotomus* are classified into five subgenera: *Phlebotomus* Rondani, 1843, *Paraphlebotomus* Theodor, 1948, *Larroussius* Nitzulescu, 1931, *Transphlebotomus* Artemiev, 1984 recently added to the check-list due to a single record of *Phlebotomus mascittii,*, and newly established subgenus *Artemievus* Depaquit, 2021, which contains only one species, *Ph. alexandri*, previously included in *Paraphlebotomus* subgenus [10, 11]. The genus comprises the proven vectors of *Leishmania* parasites and arboviruses such as *Ph. papatasi*, *Ph. sergenti*, *Ph. perfiliewi*, and *Ph. perniciosus* [12, 13]. The *Sergentomyia* species present in this area belong to four subgenera: *Parrotomyia* Theodor 1958, *Sintonius* Nitzulescu, 1931, *Grassomyia* Theodor, 1958 (sometimes considered as a genus), and *Sergentomyia* França and Parrot 1919 [14, 15]. *Sergentomyia* species are expected to feed mainly on cold-blooded vertebrates and therefore their epidemiological role and involvement in the transmission of *Leishmania* parasites and arboviruses remain unclear [15], even though some studies reported the natural infection of some species with *Leishmania* promastigotes and Toscana virus [16, 17].

The taxonomy of sand flies is traditionally based on decisive morphological characters present on the head (cibarium and pharynx) and genitalia (spermathecae for the females, whole genitalia for males). Their assessment remains the golden standard method used to describe and identify species. However, conclusive species identification of some cryptic species that are morphologically challenging or indistinguishable requires the use of molecular methods such as DNA-based techniques to provide further resolution. Examples from the Maghreb region include *Ph. chabaudi*/*Ph. riouxi* and atypical *Ph. perniciosus*/*Ph. longicuspis* [18–20]. Besides species identification, the determination of the source of the ingested blood in hematophagous insects is an essential parameter to understanding the interaction between sand fly and hosts and their impact on the epidemiology of leishmaniasis to incriminate putative hosts as reservoirs and to design correct and efficient disease control strategies [21]. Recently, two matrix-assisted laser-desorption/ionization time of flight mass spectrometry-based methods, MALDI-TOF MS protein profiling and MALDI-TOF MS peptide mass mapping, were successfully applied in sand fly studies, showing their efficiency in (i) time and cost-effectiveness, (ii) identification and discrimination of specimens to the species level, and (iii) determination of blood meal sources even if the ingested blood load was low and/or in process of digestion [22, 23].

This study aimed to conduct an entomological survey in Ghardaïa and Illizi provinces where CL cases have been steadily increasing since 2017. To understand the role of the local sand fly species in the transmission cycles of *Leishmania*, we identified sand flies morphologically, characterized their DNA barcodes and MALDI-TOF protein profiles, determined the blood meal sources of engorged females, and screened them for *Leishmania* infection.

## Methods

### Sand fly collections

**Fig. 1:**
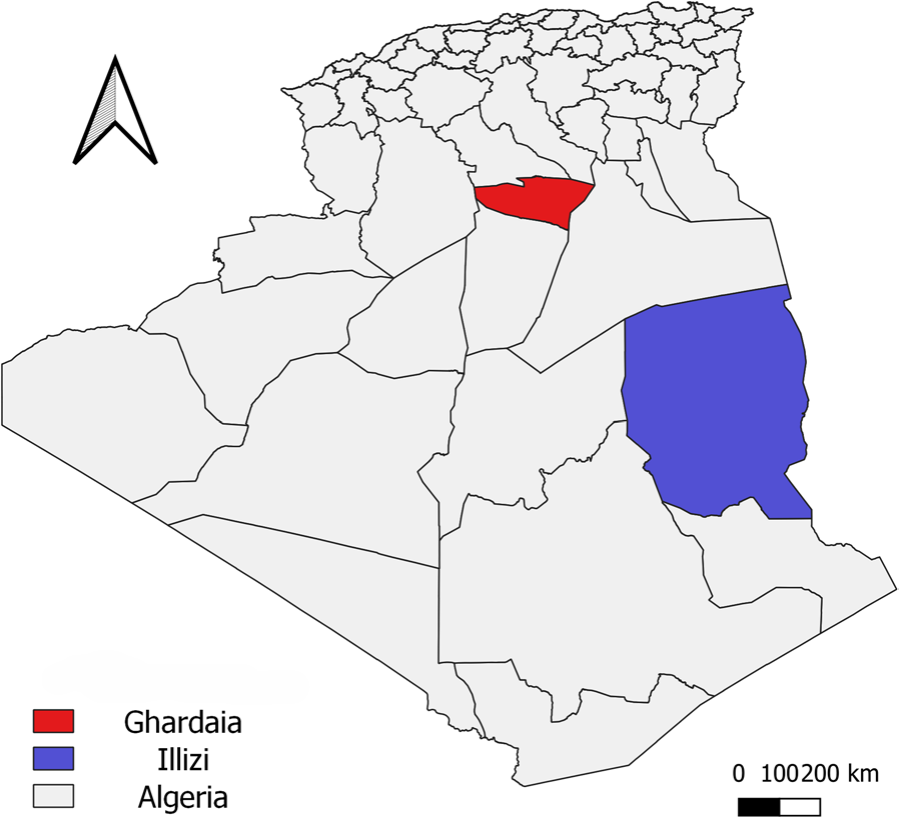
Location of the sand fly study areas

Ghardaïa and Illizi are two provinces located in north and central Sahara of Algeria, respectively (Fig. 1). They are characterized by a Saharan bioclimatic stage. Ghardaïa, located about 600 km from Algiers, is an oasis in a depression, surrounded by hills. The local human population lives in well-built houses of which some have gardens with animal shelters. Most of the households spend the summertime in their secondary houses located typically in the palmeries to avoid hot summer temperatures, thus coming in direct contact with rodents and sand flies, which increases the risk of parasite transmission. Illizi, located about 2000 km from the capital city of Algeria, is a mix of sandy and rocky regions where the human population lives mainly on pastoral activity by breeding dromedaries, sheep, and goats. The human habitations and animal shelters are huts built with mud and stones, thus providing favorable environmental conditions for sand fly breeding and development.

In June and October 2021, sand flies were trapped for 5 nights in each province using ten CDC miniature light traps (model 512 John W. Hock Company, Gainesville, FL, USA). In Illizi, the traps were set in human dwellings and animal shelters (sheep, goats, and camels), and in Ghardaïa, the traps were set in a zoo near animal shelters (chicken, birds, dogs, wolves, hyena), empty cages, and burrows in the hill surrounding the zoo. The traps were set at 2 m above the ground from sunset to sunrise. The collected sand flies were anesthetized with cigarette smoke and pooled by site and date of collection. The samples were conserved and stored in 70% ethanol at +4 °C until further analysis. The map of the study areas (Fig. 1) was performed using QGIS version 3.20.3-Odense [24].

### Morphological identification of sand flies

Head and 2–3 terminal segments of the sand fly abdomens were dissected and slide-mounted in a drop of CMCP-10 mounting medium (Polysciences, Inc., Warrington, PA, USA) for morphological identification following the identification keys described in [9, 25, 26]. The thoraxes and the rest of the abdomens were conserved in 70% ethanol at +4 °C for molecular and MALDI-TOF MS analysis. Images of the specimens and measurements of decisive morphological characters were taken and drawn using an Olympus BX51 microscope equipped with drawing tube. The morphological description of the new species was performed according to Galati et al. [27].

### DNA-based identification of sand flies

DNA was isolated from the sand fly abdomens with High Pure Template Preparation Kit (Roche, Germany) following the manufacturer’s instructions. Morphological identification was confirmed by sequencing two different targets of the mitochondrial genome: cytochrome oxidase I (Cox I) or cytochrome B (Cyt B), which was used as an alternative genetic marker in the case that Cox I gene amplification failed. Polymerase chain reaction (PCR) amplification of both markers was performed in 50 µl reaction volume using LCO 1490/HCO 2198 and the Cyt B CB3-PDR/N1N-PDR primers pairs and amplification conditions previously described [28, 29]. The amplification products were first separated and visualized on 2% agarose gel, then purified using a High Pure PCR product Purification kit (Roche). Double-stranded Sanger sequencing was performed using the same amplification primers (ABI Prism BigDye terminator Cycle Sequencing Ready Reaction Kit). The data were edited in BioEdit v.7.2.5 [30] and the generated sequences of Cox I gene were deposited in GenBank database (accession numbers given in Results). Each sequence was blasted against the GenBank database for identification. The sequence was considered correct if it matched > 98% of resemblance, *E*-value equal to 0 and rate coverage equal to 100%.

### Molecular screening for *Leishmania* infection in sand flies

Extracted DNA was either tested individually or pooled (2–12 sand flies of the same species and location per pool). The screening for *Leishmania* infection was done by the amplification of conserved sequences of the *Leishmania* kinetoplast minicircle DNA 13A/13B. The amplification reactions were carried out in a total volume of 25 µl containing 12.5 µl of Dream Taq Master Mix, 8.5 µl of deionized water, 1 µl of each primer, and finally 2 µl of DNA template and subjected to the following PCR conditions: an initial denaturation step at 94 °C for 3 min followed by 40 cycles of 94 °C for 20 s, 61 °C for 30 s, 72 °C for 7 s, and terminated with an extension step at 72 °C for 5 min [31]. Negative (DNA-free water) and positive (*L. major* DNA) controls were included for PCR assays. The amplified PCR products were separated and visualized on 2% agarose gel under a UV illuminator.

### Data analysis

The sequences were aligned using the MUSCLE alignment algorithm. In addition to our sequenced specimens, five *Se. cincta* Cox I sequences originating from Cameroon were obtained from GenBank database (MH577067, MH577068, MH577069, MH577087, MH577094) and used in the analysis. Maximum likelihood (ML) tree was built, and the nearest-neighbor interchange tree search method was performed with 1000 bootstrap replicates under assumptions of the Tamura 3-parameter model (T92 + I), the best fit evolutionary model as defined in Mega v10.1.7 [32]. The different haplotypes were inferred using DnaSp v6.12.03 [33] and parsimony network using TCS method was generated in PopArt [34]. The obtained results were also compared by ABGD using K2P distances generated through pairwise sequence comparisons using the default settings of *P*-values (0.1–0.001) in the Automatic Barcode Gap Discovery (ABGD) web-interface program (https://bioinfo.mnhn.fr/abi/public/abgd/abgdweb.html), which was used to assign each barcode sequence into molecular operational taxonomic units (MOTUs) [35]. Pairwise Kimura 2-parameters genetic distances were obtained for Cox I dataset under Mega v10.1.7 [32].

### Species identification by MALDI-TOF MS protein profiling

MALDI-TOF MS protein profiling was carried out as previously described [22]. Briefly, the thoraxes were air-dried and then manually ground in 10 μl of 25% formic acid by a BioVortexer homogenizer (BioSpec, Bartlesville, USA) using disposable pestles for 15 s. After a short centrifugation of the homogenate (10,000 × g for 15 s), 2 μl of the supernatant was mixed with 2 μl of MALDI matrix and 1 μl of this solution was deposited on a MALDI target (Bruker Daltonics, Bremen, Germany) in duplicate. The MALDI matrix was freshly prepared as an aqueous 60% acetonitrile/0.3% TFA solution of sinapinic acid (30 mg/ml; Bruker Daltonics). Protein mass spectra were measured on an AutoFlex Speed MALDI-TOF spectrometer (Bruker Daltonics) within a mass range of 3–25 kDa, calibrated externally using the Bruker Protein Calibration Standard I and visualized by FlexAnalysis v.3.4 (Bruker Daltonics). Each spectrum corresponded to an accumulation of 8000 laser shots (40 × 200 laser shots from different positions of the sample spot). For cluster analysis and species identification, the spectra were processed by MALDI Biotyper v.3.1 (Bruker Daltonics) and searched against an in-house reference database comprising reference protein profiles of 35 different sand fly species including five members of the subgenus *Sergentomyia* (*Se. antennata*, *Se. dentata*, *Se. fallax*, *Se. minuta*, and *Se. schwetzi*). Log score value (LSV) > 2.0 was considered as a threshold for unambiguous species identity assignment.

### Blood meal analysis by peptide mass mapping MALDI-TOF mass spectrometry

The air-dried abdomens were homogenized individually in 50 μl of ultra-pure water (Merck KGaA, Darmstadt, Germany) by BioVortexer homogenizer (BioSpec) for 15 s; 10 μl of the homogenate were then incubated with 10 μl of 50 mM N-ethylmorpholine acetate buffer (pH 8.1; Sigma-Aldrich) and 100 ng of trypsin (Promega) at 37 °C for 30 min. After the digestion, 0.5 μl of the mixture was deposited on a MALDI plate in duplicate, air-dried, and covered with 0.5 μl of MALDI matrix (aqueous 50% acetonitrile/0.1% TFA solution of α-cyano-4-hydroxycinnamic acid; 5 mg/ml; Bruker Daltonics). Simultaneously, the remaining 40 μL of the abdomen homogenates were utilized as a template for DNA isolation by High Pure PCR Template Preparation Kit (Roche, Germany) following the manufacturer’s protocol. The isolated DNA was screened for presence of *Leishmania* sp. in the ingested blood. Peptide mass mapping (PMM) spectra were acquired on an Ultraflex III MALDI-TOF instrument (Bruker Daltonics) in the mass range of 700–4000 Da and calibrated externally using a PepMix II peptide standard (Bruker Daltonics). For the tentative host assignment, at least two peptides per specimen were selected for MS/MS sequencing. The obtained spectra were searched against SwissProt database subset of vertebrate hemoglobins using in-house MASCOT v.2.7 search engine (Matrix Science).

## Results

### Sand fly species composition

A total of 640 sand flies were collected in the two study areas; 430 specimens from Illizi (villages: Ifni, Imoussouane, Indgidad-Imihrou, and Tihoubar-Imihrou) and 210 from Ghardaïa (districts: Ghardaïa and El Atteuf). A total of 14 different species belonging to *Phlebotomus* and *Sergentomyia* genera, previously found in Algeria, were identified in our study (Table 1). El Atteuf, Ifni, and Tihoubar were the most diversified sites with ten, seven, and six different species, respectively. In Illizi, *Ph. papatasi* was the most abundant (260 specimens), followed by *Ph. alexandri* (76 specimens), while in Ghardaïa, *Se. antennata* was the most common species (78 specimens), followed by *Se. fallax* (35 specimens). Interestingly, one female specimen collected in Illizi belonged to the genus and subgenus *Sergentomyia* but showed unique morphological features that could not be attributed to any described species so far.

**Table 1:** Sand flies from Illizi and Ghardaïa identified by morphological and molecular assessment

Province	Region	Genus	Subgenus	Species	Male	Female	Total
Illizi	Ifni (25.77069°N, 7.93326°E)	*Phlebotomus*	*Phlebotomus*	*papatasi*	2	8	10
				*bergeroti*	8	3	11
			*Larroussius*	*perniciosus*	0	1	1
			*Artemievus*	*alexandri*	41	27	68
		*Sergentomyia*	*Sergentomyia*	*antennata*	13	13	26
			*Sintonius*	*clydei*	4	14	18
				*christophersi*	6	1	7
	Indgidad-Imihrou (25.80916° N, 8.68107° E)	*Phlebotomus*	*Phlebotomus*	*papatasi*	1	12	13
			*Artemievus*	*alexandri*	1	2	3
		*Sergentomyia*	*Sintonius*	*clydei*	0	2	2
	Imoussouane (26.38665°N, 8.54841°E)	*Phlebotomus*	*Phlebotomus*	*papatasi*	51	117	168
		*Sergentomyia*	*Sergentomyia*	*schwetzi*	1	4	5
			*Sintonius*	*clydei*	6	12	18
	Tihoubar-Imihrou (25.94156°N, 8.72736°E)	*Phlebotomus*	*Phlebotomus*	*papatasi*	7	62	69
			*Artemievus*	*alexandri*	0	5	5
		*Sergentomyia*	*Sergentomyia*	*antennata*	1	0	1
				*imihra* n. sp.	0	1	1
			*Sintonius*	*clydei*	1	1	2
				*christophersi*	2	0	2
		Total			145	285	430
Ghardaïa	Ghardaïa (32.511310°N, 3.627452°E)	*Phlebotomus*	*Phlebotomus*	*papatasi*	1	0	1
		*Sergentomyia*	*Sergentomyia*	*minuta*	1	0	1
				*fallax*	2	0	2
			*Sintonius*	*clydei*	1	0	1
	El Atteuf (32.439935°N, 3.715423°E)	*Phlebotomus*	*Phlebotomus*	*papatasi*	20	7	27
			*Larroussius*	*perniciosus*	1	5	6
				*longicuspis*	4	2	6
			*Paraphlebotomus*	*sergenti*	30	0	30
		*Sergentomyia*	*Sergentomyia*	*antennata*	58	20	78
				*fallax*	21	12	33
				*minuta*	1	2	3
			*Sintonius*	*clydei*	12	4	16
				*christophersi*	5	0	5
			*Grassomyia*	*dreyfussi*	0	1	1
		Total			157	53	210

### Sequencing analysis

**Fig. 2:**
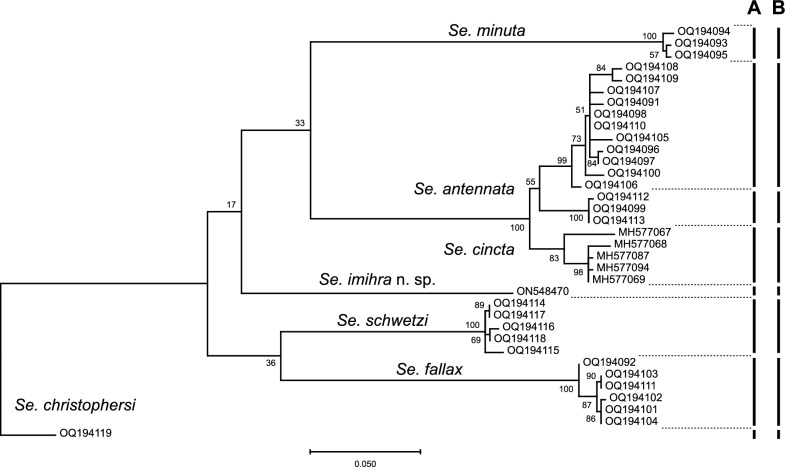
Maximum likelihood tree of *Sergentomyia* Cox I gene, 1000 bootstrap replicates. **A** ABGD result, **B** TCS haplotype network

PCR amplification of Cox I was successful for all studied species except for *Se. clydei*, for which the amplification attempts of the Cox I gene repeatedly failed for all analyzed specimens. Cyt B gene was successfully amplified for the unknown *Sergentomyia* female in addition to *Se. clydei*, for which Cox I amplification failed. Cox I generated sequences of 680 pb for all the species except *Se. christophersi* (661 bp) and *Se. imihra* (687 bp). After alignment and trimming of the sequences generated from the specimens belonging to the *Sergentomyia* subgenus, a ML tree was built using the Cox I marker to solve the identity of the new *Sergentomyia* specimen (Fig. 2). *Se. christophersi* (OQ194119) was used as an outgroup. The ML tree showed the presence of six distinct clusters: *Se. minuta* (OQ194093 to OQ194095), *Se. fallax* (OQ194092, OQ194101 to OQ194104, OQ194111), *Se. schwetzi* (OQ194114 to OQ194118), *Se. antennata* (OQ194091, OQ194096 to OQ194100, OQ1940105 to OQ194110, OQ194112, OQ194113), *Se. cincta* (MH577067, MH577068, MH577069, MH577087, MH577094), and *Sergentomyia* sp. (ON548470), which we further characterized and formally described as *Sergentomyia imihra* n. sp. The ABGD software using the prior intraspecific divergence threshold value of 1.29% showed the same result (Additional file 1: Fig. S1) and resolution of the species order in the phylogenetic tree (Fig. 2A). Furthermore, the haplotype analysis (Additional file 2: Fig. S2) revealed the presence of 29 different haplotypes (haplotype diversity, *Hd* = 0.9911). The TCS network showed the presence of six haplogroups: *Se. minuta*, *Se. fallax*, *Se. schwetzi*, *Se. antennata*, *Se. cincta*, and *Se*. *imihra* n. sp. Interestingly, the TCS tree also supported the validity of *Se. imihra* n. sp., which was branched between *Se. fallax* and *Se. antennata*, 61 substitutions discriminating it from *Se. antennata*, 70 from *Se. fallax*, 68 from *Se. cincta*, 127 from *Se. minuta*, and 138 from *Se. schwetzi*. The results (Fig. 2B) confirmed its status as a new species and not a morphological malformation often reported within the *Sergentomyia* genus. The genetic distance between *Se. imihra* n. sp. and the rest of the subgenus *Sergentomyia* species varied between 0.160 and 0.189 (Additional file 3: Table S1). Furthermore, congruent assignment of species was seen between ABGD, TCS network, and ML tree. In addition, *Se. antennata* specimens, which were grouped in a single clade in ML analysis while they were assigned to two clusters by ML tree, ABGD, and TCS network, showed a genetic distance ranging from 0.037 to 0.056 between three specimens: OQ194099, OQ194113 (females trapped in Illizi), and OQ194112 (male trapped in Ghardaïa) and the rest of *Se. antennata* (Additional file 4: Table S2). The statistical analysis performed in DnaSP using Fu and Li’s D* = 0.20764 (*P* > 0.10) and Fu and Li’s F* = 0.10483 (*P* > 0.10) [36] did not show significant difference among analyzed *Se. antennata* specimens. Similarly, morphological identification and MALDI-TOF MS protein profiling did not show any difference.

### Molecular screening for *Leishmania* infection in sand flies

A total of 112 females (53 form Ghardaïa, 59 from Illizi) were subjected to *Leishmania* screening; 47 were engorged and 65 specimens were unfed. The results showed that all analyzed females were negative for *Leishmania* parasites (Additional file 5: Table S3).

### Species identification by MALDI-TOF MS protein profiling

**Fig. 3:**
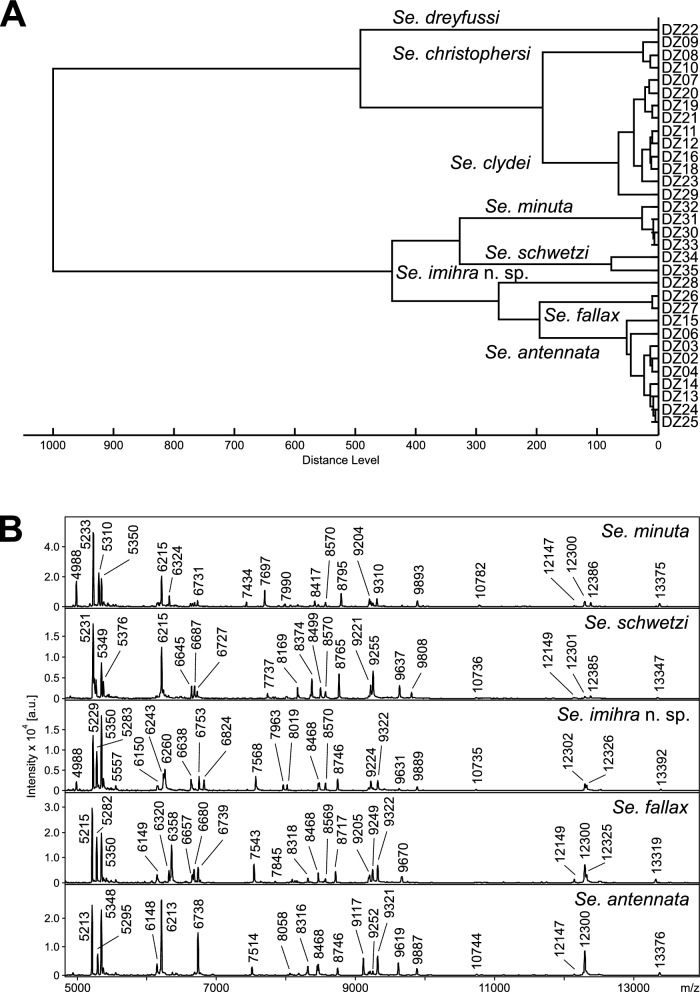
**A** Dendrogram of MALDI-TOF MS protein profiles of eight *Sergentomyia* species collected in Algeria. The distances are in relative units. **B** Comparison of mass spectra of *Se. imihra* n. sp. with four other members of the *Sergentomyia* subgenus found in the Central Sahara of Algeria

**Fig. 4:**
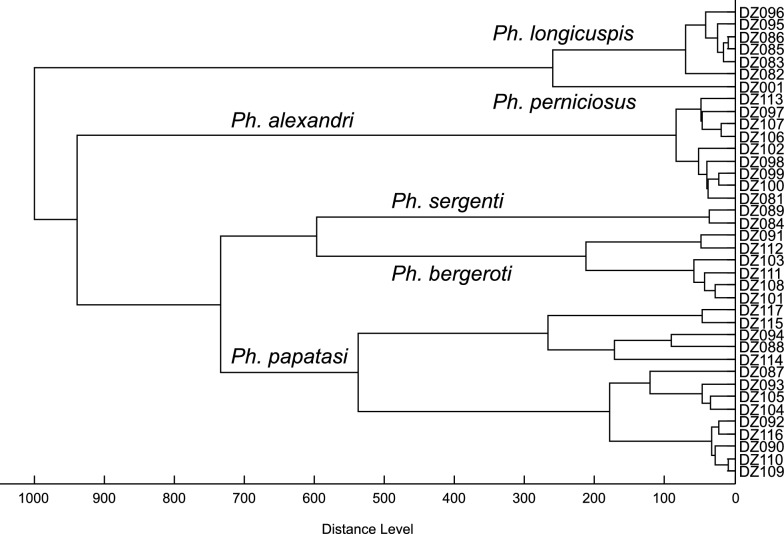
Dendrogram of MALDI-TOF mass spectra of six *Phlebotomus* species collected in Algeria. The distances are shown in relative units

Of all trapped sand flies, 110 specimens were subjected to MALDI-TOF MS analysis either for species identification (72) or blood meal origin determination (38); 29 of those 72 specimens were identified by both morphology and DNA sequencing, and 33 were assigned solely by morphology (Additional file 6: Table S4). The remaining ten specimens were not reliably identified either by molecular analysis or morphology due to missing or poorly visible decisive morphological characters or failure of PCR amplification or sequencing. Of 72 specimens analyzed by MALDI-TOF protein profiling, 70 provided high-quality spectra suitable for further evaluation. Only two specimens (DZ36, DZ38) gave spectra of poor quality, yielding no species identification. The protein profiles of some sand flies conclusively determined by DNA sequencing and/or morphologically were used to upgrade our in-house reference database [*Se. fallax* (2), *Se. antennata* (9), *Se. dreyfussi* (1), *Se. imihra* n. sp. (1), *Se. christophersi* (3), *Se. clydei* (9), *Ph. alexandri* (9), *Ph. bergeroti* (6)]. The species assignment obtained by comparison of protein profiles with the upgraded database nicely matched with the species identity of 60 specimens uncovered earlier by DNA sequencing and/or morphologically. The protein profiling further corrected the species determination of five specimens—DZ11, DZ12, DZ23, DZ29, and DZ81—and confirmed DNA sequencing result of DZ24 and DZ25, which were incorrectly determined by morphology as *Se. fallax* (Additional file 6: Table S4). Interestingly, the result of protein profiling was also congruent with the molecular identification of the novel species *Se. imihra* n. sp., which formed a separate branch that was distinct from other analyzed species of *Sergentomyia* subgenus and positioned close to the clusters of *Se. antennata* and *Se. fallax* in the dendrogram (Fig. 3A). In addition, the protein profile of *Se. imihra* n. sp. was obviously different from the spectra of four other members of *Sergentomyia* subgenus showing a number of species-specific peaks (Fig. 3B). This technique further supported the proposition of including *Ph. alexandri* into the new subgenus *Artemievus* [11]. In the dendrogram, all *Ph. alexandri* specimens grouped unequivocally into a single and distant cluster from *Ph. sergenti* (Fig. 4), which belongs to *Paraphlebotomus* subgenus. The appearance of two branches for *Ph. papatasi* could be explained by the compromised spectra quality of some specimens.

### Blood meal analysis by PMM-based MALDI-TOF mass spectrometry

A total of 38 blood-fed sand fly females (4 from Ghardaïa and 34 from different villages of Illizi) were analyzed individually using PMM-based MALDI-TOF mass spectrometry to determine the origin of their blood meals. Before PMM analysis, the engorged females were taxonomically classified using decisive morphological characters [9], resulting in four different species: *Ph. papatasi* (31), *Ph. alexandri* (5), *Ph. longicuspis*, (1) and *Ph. perniciosus* (1). The PMM-based MALDI-TOF MS approach successfully determined blood meal origin in 87% (33/38) of the engorged females (Additional file 7: Table S5). For five samples, the analysis did not provide any host identification, probably due to the small volume of blood taken or the advanced blood digestion. However, the majority of the analyzed females yielded spectra of high quality, allowing for conclusive blood meal source assignment. Altogether, eight different hosts were identified: camel (*Camelus dromedarius*), dog (*Canis lupus familiaris*), sheep (*Ovis aries)*, goat (*Capra hircus*), chicken (*Gallus gallus*), human (*Homo sapiens*), donkey (*Equus asinus*), and horse (*Equus caballus*). The most numerous blood meal source (Additional file 8: Table S6) was goat (13), followed by camel, dog, and human (5); sheep and donkey (2); and finally, chicken and horse (1). In all successfully identified samples, *Ph. papatasi* showed opportunistic behavior with six different hosts and one mixed blood meal (human + goat) followed by *Ph. alexandri* with five different hosts. Only one blood-fed female of *Ph. perniciosus* and one *Ph. longicuspis* were collected and analyzed, both blood meals identified as originating from a donkey.

### Morphological description of *Sergentomyia imihra* n. sp.

The evaluation of the decisive morphological characters as well as findings of molecular analyses described above suggest the presence of a new species belonging to the genus *Sergentomyia* França and Parrot, 1920, and subgenus *Sergentomyia*, defined by having (i) recumbent hairs on the abdominal tergites, (ii) tubular spermathecae with smooth walls of uniform width along their length, and (iii) antennal segment 3 is shorter than segments 4 and 5 together and usually shorter than the labrum [14, 25, 37].

**Family Psychodidae Newman, 1834**

**Genus** ***Sergentomyia*** **França and Parrot, 1920**

**Subgenus** ***Sergentomyia***

**Species** ***Sergentomyia imihra*** **n. sp. Benallal, Depaquit and Dvořák, 2024**

***Type locality***: Tihoubar-Imihrou (25°0.94365N, 8°0.72767 E) is a rocky region located in south of the province of Illizi, central Sahara, South-Eastern Algeria.

***Type material***: Holotype female has been deposited in the Institut Pasteur of Algeria (IPA) under identification number 472.

***Representative DNA sequences***: GenBank accession numbers: ON548470 (Cox I) and OQ190728 (Cyt B).

***ZooBank registration***: To comply with the regulations set out in Article 8.5 of the amended 2012 version of the International Code of Zoological Nomenclature (ICZN) [38], details of the new species have been submitted to ZooBank. The Life Science Identifier (LSID) of the article is urn:lsid:zoobank.org:pub:673E442C-ED4E-4C44-942C-41FF8BD6FF00. The LSID for the new species *Sergentomyia imihra* is urn:lsid:zoobank.org:act:C420A58D-8A10-4348-AE33-E524E2A13594.

***Note*:** The authors of the new taxa are different from the authors of this paper: Article 50.1 and Recommendation 50A of the International Code of Zoological Nomenclature.

***Etymology***: The species is dedicated to the village where the specimen was trapped.

### Description

**Fig. 5:**
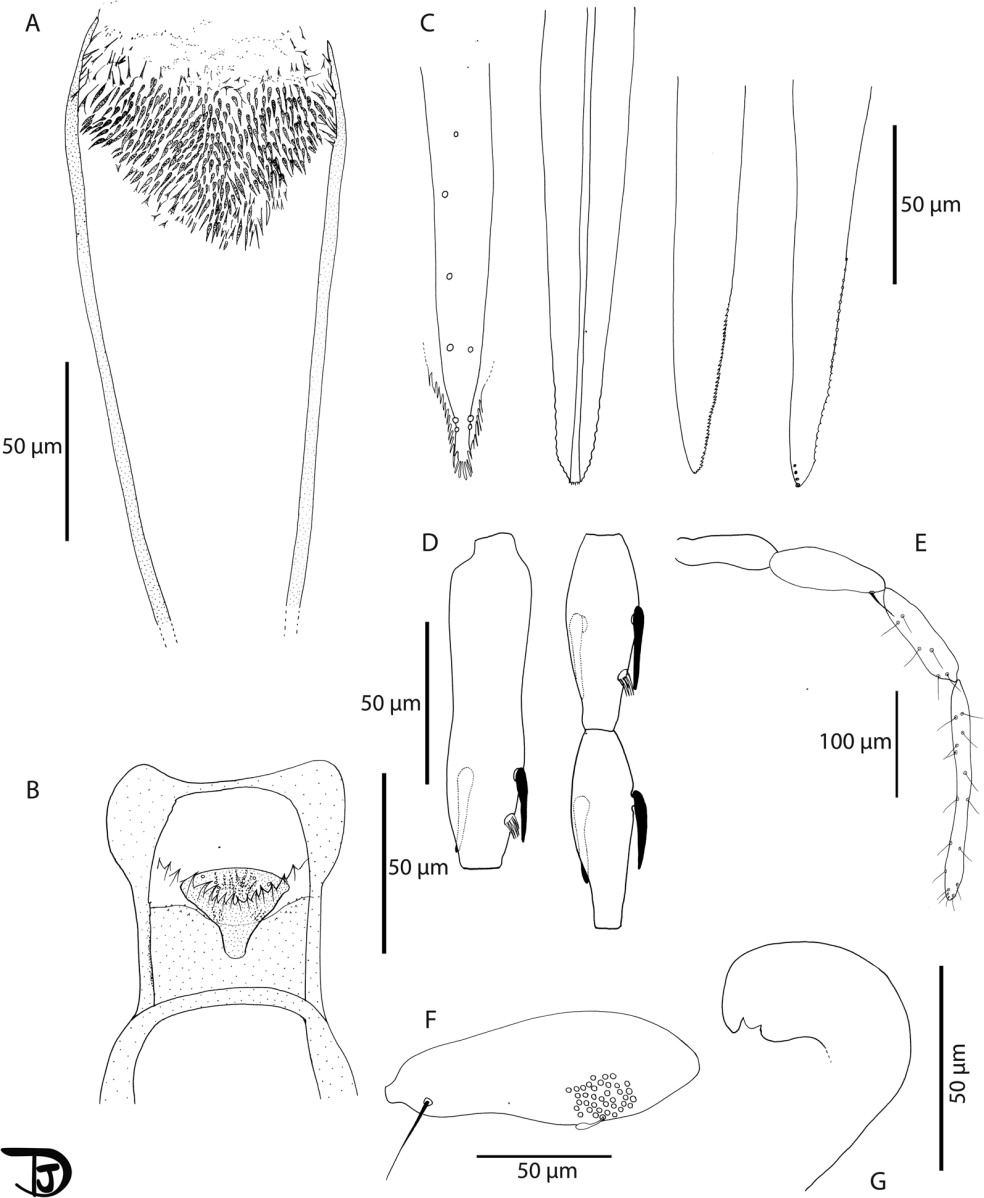
Morphological description of *Sergentomyia imihra* n. sp. female. **A** Pharynx. **B** Cibarium. **C** Mouthparts (labrum, hypopharynx, mandible, and maxilla). **D** Flagellomeres 1, 2, and 3. **E** Labium: palpi. **F** Detail of the third article of the palpi. **G** Spermatheca

***Female***. The counts and measurements provided below are those of the holotype labeled *Se.* 472 and deposited at IPA (Fig. 5, Additional file 9: Fig. S3) *Head*. Occiput with two narrow lines of well-individualized setae. On the line above the eyes, insertion of seta on each side. Clypeus 115 μm long with 30 setae randomly distributed. Eyes 221 μm long, 148 μm wide. Interocular sutures complete reaching the interantennal one. Flagellomeres: f1 (= AIII) = 100 μm shorter than f2 (= AIV) = 57 μm + f3 (= AV) = 58 μm, f12 (= AXIV) = 40 µm, f13 (= AXV) = 36 µm and f14 (= AXVI) = 50 µm. Ascoidal formula: 2/f1-f11 & 1/f12-f13 with short ascoids, covering three-fourths of the article. One papilla one flagellomeres 1 and 2. Absence of papilla on the third flagellomere. Palpi: p1 = 20 μm long; _*P*_2 = 70 μm; _*P*_3 = 112 μm; _*P*_4 = 105 μm, _*P*_5 = 197 μm. Palpal formula: 1, 2, 4, 3, 5. About 38 Newstead’s sensilla are visible in the third palpal segment, part of a larger group. No Newstead’s sensilla on other palpal segment. Labrum-epipharynx 160 μm long. f1/L = 0.625. Maxillary lacinia exhibiting 4 external teeth and 30 fine internal teeth. More than 50 teeth on the mandible. Hypopharynx with a dozen of wavy teeth. Cibarium is covered with a small heterogeneously pigmented sclerotised area, and one row of 16 concave sharply pointed teeth oriented backward. Pharyngeal armature is well developed, occupying the last third of the pharynx, made with large teeth oriented forward. *Thorax, wings, abdomen, and legs*. Not observed due to molecular processing. *Genitalia*. Spermathecae are tubular with smooth and thin walls of uniform width along their length, length = 95 μm, width = 17 μm.

Detailed morphological analysis of this new species provided morphological features distinguishing it from the phylogenetically closely related species *Se. cincta* described by Abonnenc and Parrot [25, 39], *Se. antennata*, and *Se. sintoni* [40] as summarized in Table 2.

**Table 2:** Summary of morphological differences between *Se. imihra* n. sp. and the phylogenetically closely related *Sergentomyia* species

Character	*Se. imihra* n. sp. Current study	*Se. cincta* [25]	*Se. cincta* [39]	*Se. antennata* [25]	*Se. sintoni* [40]
Ascoidal formula	2/f1–f11 and 1/f12–f13	*2/f1–f13*	*2/f1–f13*	*2/f1–f13*	N/I
Flagellomere	f1:100 μm	*f1:78–88 µm*	f1:105–120 µm	f1: 80–100 µm	*f1:142–152 µm*
Palpal formula	p1: 20 μm; p2: 70 μm; p3: 112 μm; p4:105 μm; p5:197 μm Total length: 504 µm	Total length: 490–510 µm	*Total length: 205 µm*	Total length: 560 µm	N/I
Cibarium	16 sharply pointed teeth and about 4–6 denticules on each side	14–18 short sharply pointed teeth and about 5–8 denticules on each side	16–20 sharply pointed teeth and about 5–6 denticules on each side	*22–34 sharply pointed, mono-morph teeth displayed on a backward concave shape, covered with a triangular pigmented patch area, including one row of 4–6 denticules on each side*	*10–14 pointed teeth, without denticles*
Pharyngeal armature	Well developed, occupying the last third of the pharynx, displaying sharp and large teeth	*Helmet-like shape, anterior pharynx about three times larger backward than forward carrying strong and tight brush-like spines *	*Heart-shaped, three times larger backward than in front*	*Large backward and displayed rounded and strong denticules*	*Heart-shaped*

Distinguishing characters are in italics. N/I, not indicated

## Discussion

Sand fly fauna in Ghardaïa and Illizi, two Algerian provinces with emerging cases of human cutaneous leishmaniasis, was described by combined morphological and molecular approaches. In total, we recorded 14 sand fly species, 6 belonging to *Phlebotomus* genus and 8 *Sergentomyia* genus. Species composition and abundance of recorded species differed among the two studied foci. All identified species of the genus *Phlebotomus*, belonging to four different subgenera, are known to be either proven or potential vectors of several *Leishmania* species that are causative agents of human leishmaniases [41]. In our study, the PCR screening for *Leishmania* spp. was negative for the proven vectors of *Ph. papatasi*, *Ph. sergenti*, and *Ph. perniciosus* [6, 42, 43], as well as for all screened species of the genus *Sergentomyia* expected to transmit *Sauroleishmania* spp. [44]. Vector competence of *Sergentomyia* species for human parasites remains hypothetical. Thus far, performed assays deploying experimental infections disapproved this hypothesis using specimens from laboratory colonies [45, 46]. However, the vast majority of species of the genus *Sergentomyia* cannot be bred in captivity, hence making the experimental testing of their vector competence impossible [47]. *Sergentomyia minuta* is suspected to play a role in the epidemiology of leishmaniasis and arboviruses in the African continent in relation to its distribution and feeding habits [16, 17].

We recorded eight species of the *Sergentomyia* genus, which is known as the most diversified among Old World sand flies, harboring 276 species [14]. In Algeria, 12 species of the genus have been recorded [9]; *Se. hirtus* is the last described species in the central Sahara [48] but its morphological features are sometimes regarded as a malformation [26]. Recent systematics of this genus recognizes seven subgenera on the basis of their spermathecal morphology: *Sergentomyia* with smooth, thin-walled, and wide spermathecae; *Sintonius* Nitzulescu, 1931; *Trouilletomyia* Depaquit and Léger, 2014, with annealed spermathecae; *Parrotomyia* Theodor, 1958, with elliptical capsule, smooth, thin, or thick-walled spermathecae; *Rondanomyia* Theodor, 1958, with smooth and wide spermathecae; *Capensomyia* Davidson, 1979, with convoluted spermathecae; and *Vattieromyia* Depaquit, Léger, and Robert, 2008. Subgenus *Grassomyia* Theodor, 1958, which exhibits round, sclerotized capsules with small spicules spermathecae, is considered by some authors as a genus [14].

Our complex effort enabled recognition and formal description of a new species *Sergentomyia (Sergentomyia) imihra* n. sp. Benallal, Depaquit and Dvořák, 2024, within the subgenus *Sergentomyia*, as suggested by presence of smooth spermathecae [14, 25]. Unique identification of the newly described species, its classification and distinction from other species of *Sergentomyia* subgenus, has been successful thanks to a combination of morphological and molecular tools as recently suggested in the description of *Phlebotomus betisi* and the new subgenus *Lewisius* [49]. Moreover, the species status of *Se. imihra* n. sp. is supported by sufficiently divergent sequences of mitochondrial Cox I and Cyt B genes recommended as molecular markers to confirm the species identification [29, 50]. Blast analysis of Cox I sequence in GenBank showed that *Se. imihra* n. sp. presented 89.25% and 88.66% of similarity with *Se. punjabensis* and *Se. inermis*, respectively, while the CytB sequence showed 91.35% and 94.61% of similarity with *Se. dubia* and *Se. sintoni*, respectively. Although the bootstrap values in the ML tree were quite low, the genetic distance, which is high between *Se. imihra* n. sp. and the rest of the analyzed species (16.8–18.9%), the clear morphological differences, and also unique and species-specific protein profile distinct from other analyzed members of the *Sergentomyia* subgenus support that the newly described sand fly species conclusively differs from the known species of the subgenus *Sergentomyia* thus far.

During the last decade, MALDI-TOF MS protein profiling has become a method of choice for conclusive, rapid, and cost-effective species identification of various arthropod vectors including sand flies [22, 23], especially suitable for field-collected specimens from large-scale entomological surveys. In the current study, this mass-spectrometry-based technique provided high-quality and reproducible spectra yielding reliable species assignment of all except two analyzed specimens, while allowing for parallel application of other complementary approaches (DNA barcoding, morphological analysis) and screening for sand fly-borne pathogens. As was recently demonstrated for *Ph. creticus* and two other sand fly species from Laos [49, 51], MALDI-TOF MS protein profiling represents a useful tool in integrative taxonomy, and in this study supported the formal description of the newly discovered species *Se. imihra* n. sp. and further elucidated taxonomical classification of other species. Albeit not always reflecting the phylogenetic relationships within the subgenera, as shown by clustering of *Ph. sergenti* and *Ph. bergeroti* groups, the method also supported the inclusion of *Ph. alexandri* into the new subgenus [11].

Blood meal analysis is one of key steps to correctly understand trophic inclinations of various hematophagous insects, their relationship with hosts, and their roles in the eco-epidemiology of the zoonotic diseases that they transmit. Therefore, a wide array of different methods including precipitin tests, enzyme-linked immunosorbent assay (ELISA), and sequencing of mitochondrial and nuclear DNA markers were deployed to study the origin of blood meal sources [52–56]. However, success of host blood identification is challenged by various factors such as the low amount of ingested blood, its advanced digestion, possible contamination of samples, and varying time, labor, and cost investments of the analysis. Recently, PMM-based MALDI-TOF mass spectrometry allowed for a significant reduction in cost and time by a simple sample preparation, and also showed its efficacy in the determination of the blood sources, even for mixed origin, advanced blood digestion, and low amount of blood [23, 57]. In our study, this technique provided a high rate of reliable identifications and even enabled the detection of mixed blood meal for one *Ph. papatasi* specimen. We conclude that this species as well as *Ph. alexandri* can be both zoophilic or anthropophilic, suggesting that the opportunistic behavior depends on the presence of different hosts and their proximity to breeding sites. The identification of several mammalian blood sources such as camel, sheep, goat, donkey, horse, and chicken in the study areas suggests potential zooprophylactic barrier reducing human–vector contact as previously reported in the northern part of Algeria, where cattle was the main blood source identified [57]. This shall be considered for potential vector control measures and transmission risk assessment.

## Conclusions

This study provided new data on sand fly fauna in Ghardaïa and Illizi, regarding the species composition and the trophic relationships of hematophagous females with their animal hosts. Integrative approach that combined morphological analysis, sequencing of DNA markers, and protein profiling enabled the recognition and description of a new *Sergentomyia* species, raising the number of the Algerian sand fly fauna to 27 species. Further sand fly surveillance in the central Sahara is recommended to identify the thus-far unknown males of *Se. imihra* n. sp., to further elucidate the extent of the species presence in the central Sahara, and search for natural infections by *Leishmania* sp. within the sand fly fauna to understand the vector involved in the parasite transmission in the foci of emerging importance.

## Supplementary Information

Additional file 1: **Figure S1.** MOTUs inferred from ABGD software.

Additional file 2: **Figure S2.** TCS haplotype network for 34 *Sergentomyia* specimens using Cox I sequences. Circle size and color indicate frequency and species of haplotypes, respectively. Haplotype numbers are written next to the corresponding circle, dashes between haplotypes indicate mutation sites.

Additional file 3: **Table S1.** Genetic distances of Cox I sequences between Algerian *Sergentomyia* groups estimated using Tamura 3-parameter model. The number of base substitutions per site from averaging over all sequence pairs between groups are shown. The rate variation among sites was modeled with a gamma distribution.

Additional file 4: **Table S2.** Genetic distances of Cox I sequences of Algerian *Sergentomyia* species estimated using Tamura 3-parameter model. The number of base substitutions per site from between sequences are shown. The rate variation among sites was modeled with a gamma distribution.

Additional file 5: **Table S3. **Sand fly species tested for *Leishmania* spp. parasites.

Additional file 6: **Table S4. **List of sand fly specimens analyzed by MALDI-TOF MS protein profiling.

Additional file 7: **Table S5. **Peptide mass mapping MALDI-TOF MS identification of blood meal origin of engorged females.

Additional file 8: **Table S6. **Result summary of blood meal analysis using PMM-MALDI-TOF mass spectrometry.

Additional file 9: **Figure S3.** Morphological features of *Sergentomyia imihra* n. sp. female  **A** Pharynx **B** Cibarium **C** Flagellum.

## Data Availability

All data generated or analyzed during this study are included in this article and its additional files. Cox I and Cyt B sequences of sand fly specimens analyzed in this study were deposited in GenBank database under accession numbers given above. Holotype female *Se. imihra* n. sp. has been deposited in the Institut Pasteur of Algeria (IPA) as described above.

## Abbreviations

ABGD: Automatic barcode gap discovery
Cox I: Cytochrome *c* oxidase subunit 1
Cyt B: Cytochrome B
CL: Cutaneous leishmaniasis
IPA: Institut Pasteur of Algeria
K2P: Kimura two-parameter model
MALDI-TOF MS: Matrix-assisted laser desorption/ionization time-of-flight mass spectrometry
ML: Maximum likelihood
PMM: Peptide mass mapping
TFA: Trifluoroacetic acid
VL: Visceral leishmaniasis
