# Supplementary figures and images for: Phlebotomine sand fly survey, blood meal source identification, and description of Sergentomyia imihra n. sp. in the central Sahara of Algeria
Source: Parasit Vectors. 2024 Nov 4;17:449. doi: 10.1186/s13071-024-06542-9 (PMC11536750; doi:10.1186/s13071-024-06542-9)

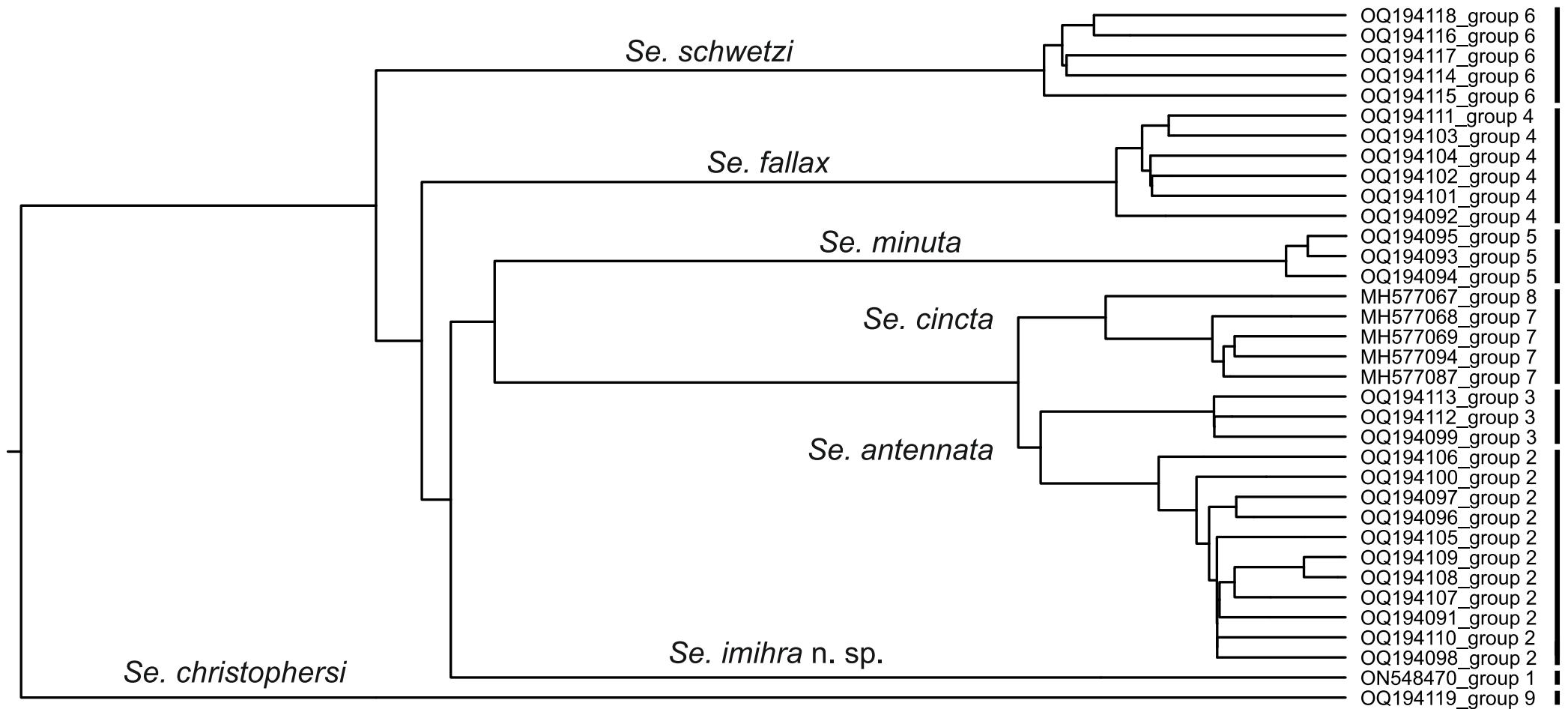

0.02

Supplement: Supplementary file 1 — Additional file 1: Figure S1. MOTUs inferred from ABGD software. [file 13071_2024_6542_MOESM1_ESM.pdf]

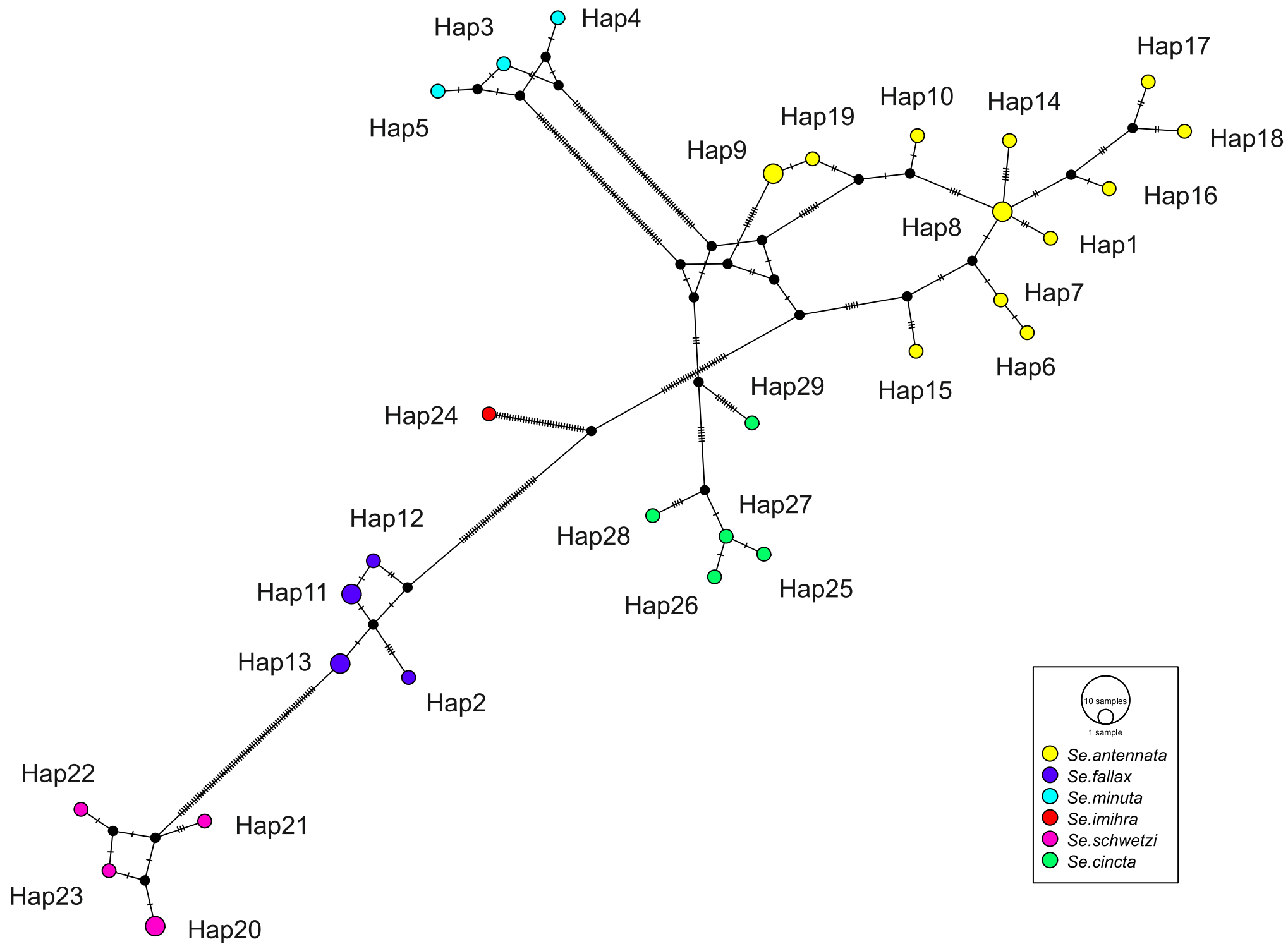

Supplement: Supplementary file 2 — Additional file 2: Figure S2. TCS haplotype network for 34 Sergentomyia specimens using Cox I sequences. Circle size and color indicate frequency and species of haplotypes, respectively. Haplotype numbers are written next to the corresponding circle, dashes between haplotypes indicate mutation sites. [file 13071_2024_6542_MOESM2_ESM.pdf]

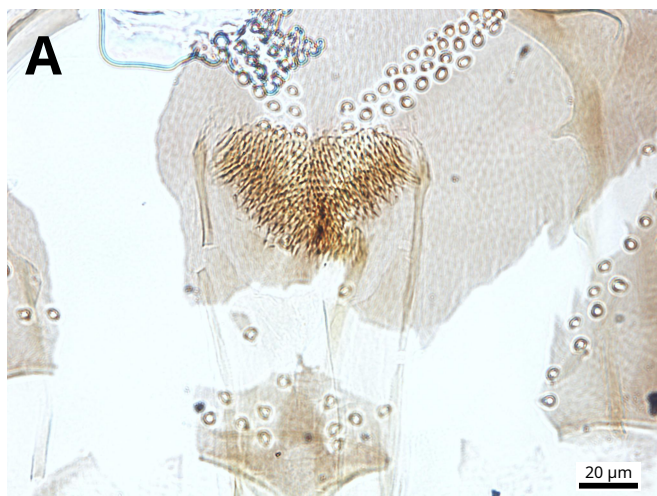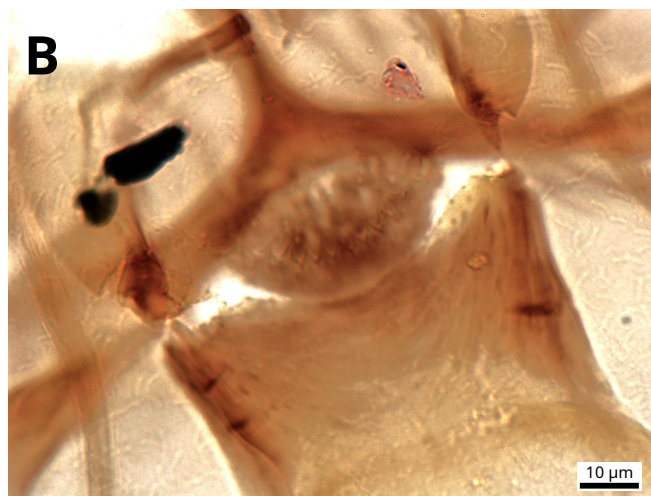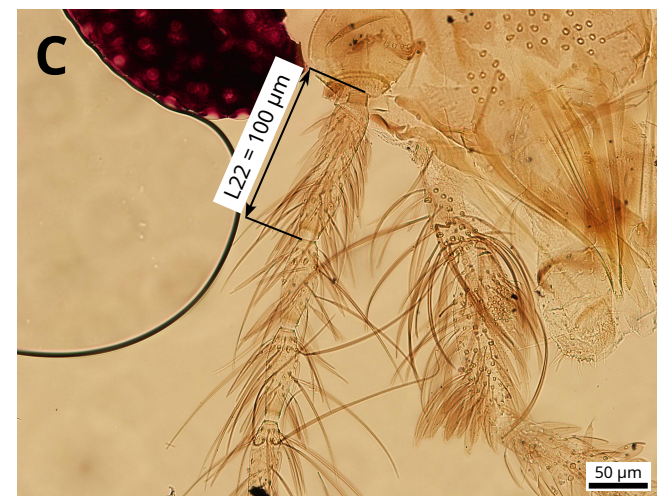

Supplement: Supplementary file 9 — Additional file 9: Figure S3. Morphological features of Sergentomyia imihra n. sp. female A Pharynx B Cibarium C Flagellum. [file 13071_2024_6542_MOESM9_ESM.pdf]
