# Supplementary material for: Phlebotomine sand fly survey, blood meal source identification, and description of Sergentomyia imihra n. sp. in the central Sahara of Algeria
Source: Parasit Vectors. 2024 Nov 4;17:449. doi: 10.1186/s13071-024-06542-9 (PMC11536750; doi:10.1186/s13071-024-06542-9)
Supplement: Supplementary file 3 — Additional file 3: Table S1. Genetic distances of Cox I sequences between Algerian Sergentomyia groups estimated using Tamura 3-parameter model. The number of base substitutions per site from averaging over all sequence pairs between groups are shown. The rate variation among sites was modeled with a gamma distribution. [file 13071_2024_6542_MOESM3_ESM.pdf]

| | [1] | [2] | [3] | [4] | [5] | [6] |
|---|---|---|---|---|---|---|
| Se. imihra [1] | | | | | | |
| Se. antennata [2] | 0.163 | | | | | |
| Se. fallax [3] | 0.163 | 0.175 | | | | |
| Se. minuta [4] | 0.189 | 0.182 | 0.188 | | | |
| Se. schwetzi [5] | 0.168 | 0.191 | 0.163 | 0.185 | | |
| Se. cincta [6] | 0.165 | 0.053 | 0.187 | 0.182 | 0.176 | |
| Se. christophersi [7] | 0.160 | 0.191 | 0.183 | 0.177 | 0.169 | 0.172 |
