# Supplementary material for: Phlebotomine sand fly survey, blood meal source identification, and description of Sergentomyia imihra n. sp. in the central Sahara of Algeria
Source: Parasit Vectors. 2024 Nov 4;17:449. doi: 10.1186/s13071-024-06542-9 (PMC11536750; doi:10.1186/s13071-024-06542-9)
Supplement: Supplementary file 5 — Additional file 5: Table S3. Sand fly species tested for Leishmania spp. parasites. [file 13071_2024_6542_MOESM5_ESM.docx]

**Additional file 5: Table S3.** Sand fly species tested for *Leishmania* spp. parasites.

| Province | Species | Engorged | Unfed |
| --- | --- | --- | --- |
| Ghardaïa | *Ph. papatasi* | 2 | 5 |
|  | *Ph. perniciosus* | 1^#^ | 4 |
|  | *Ph. longicuspis* | 1^#^ | 1^#^ |
|  | *Se. dreyfussi* | / | 1^#^ |
|  | *Se. minuta* | / | 2 |
|  | *Se. clydei* | / | 4 |
|  | *Se. fallax* | / | 12 |
|  | *Se. antennata* | / | 20 |
| Illizi | *Ph. papatasi* | 38 | 2 |
|  | *Ph. bergeroti* | / | 3 |
|  | *Ph. alexandri* | 5 | 7 |
|  | *Se. fallax* | / | 2 |
|  | *Se. antennata* | / | 2 |
| Total | | 47 | 65 |

^#^: Analysed individually
