# Supplementary material for: Phlebotomine sand fly survey, blood meal source identification, and description of Sergentomyia imihra n. sp. in the central Sahara of Algeria
Source: Parasit Vectors. 2024 Nov 4;17:449. doi: 10.1186/s13071-024-06542-9 (PMC11536750; doi:10.1186/s13071-024-06542-9)
Supplement: Supplementary file 6 — Additional file 6: Table S4. List of sand fly specimens analyzed by MALDI-TOF MS protein profiling. [file 13071_2024_6542_MOESM6_ESM.docx]

**Additional file 6: Table S4.** List of sand fly specimens analysed by MALDI-TOF MS protein profiling.

| Code | Morphological ID | DNA sequencing | MALDI-TOF MS | LSV |
| --- | --- | --- | --- | --- |
| DZ01 | *Ph. perniciosus* F | *Ph. perniciosus* | *Ph. perniciosus* | 2.378 |
| DZ02 | *Se. antennata* F | / | *Se. antennata*^#^ | 2.818 |
| DZ03 | *Se. antennata* F | / | *Se. antennata*^#^ | 2.799 |
| DZ04 | *Se. antennata* F | / | *Se. antennata*^#^ | 2.803 |
| DZ06 | *Se. antennata* M | / | *Se. antennata*^#^ | 2.728 |
| DZ07 | *Se. clydei* F | *Se. clydei* | *Se. clydei*^#^ | 2.714 |
| DZ08 | *Se. christophersi* M | *Se. christophersi* | *Se. christophersi*^#^ | 2.848 |
| DZ09 | *Se. christophersi* M | *Se. christophersi* | *Se. christophersi*^#^ | 2.823 |
| DZ10 | *Se. christophersi* M | / | *Se. christophersi*^#^ | 2.896 |
| DZ11^$^ | *Se. christophersi* M | - | *Se. clydei*^#^ | 2.681 |
| DZ12^$^ | *Se. christophersi* M | - | *Se. clydei*^#^ | 2.781 |
| DZ13 | *Se. antennata* F | *Se. antennata* | *Se. antennata*^#^ | 2.817 |
| DZ14 | *Se. antennata* F | *Se. antennata* | *Se. antennata*^#^ | 2.831 |
| DZ15 | *Se. antennata* M | *Se. antennata* | *Se. antennata*^#^ | 2.538 |
| DZ16 | *Se. clydei* F | / | *Se. clydei*^#^ | 2.841 |
| DZ18 | *Se. clydei* M | / | *Se. clydei*^#^ | 2.746 |
| DZ19 | *Se. clydei* M | *Se. clydei* | *Se. clydei*^#^ | 2.646 |
| DZ20 | *Se. clydei* M | / | *Se. clydei*^#^ | 2.696 |
| DZ21 | *Se. clydei* M | - | *Se. clydei*^#^ | 2.641 |
| DZ22 | *Se. dreyfussi* F | / | *Se. dreyfussi*^#^ | 3.000 |
| DZ23^$^ | *Se. dreyfussi* M | - | *Se. clydei*^#^ | 2.809 |
| DZ24^$^ | *Se. fallax* F | *Se. antennata* | *Se. antennata*^#^ | 2.785 |
| DZ25^$^ | *Se. fallax* F | *Se. antennata* | *Se. antennata*^#^ | 2.794 |
| DZ26 | *Se. fallax* M | / | *Se. fallax*^#^ | 2.871 |
| DZ27 | *Se. fallax* M | / | *Se. fallax*^#^ | 2.755 |
| DZ28 | *Se. imihra* n.sp F | / | *Se. imihra* n.sp.^#^ | 3.000 |
| DZ29^$^ | *Se. lewisi* M | - | *Se. clydei* | 2.447 |
| DZ30 | *Se. minuta* F | / | *Se. minuta* | 2.508 |
| DZ31 | *Se. minuta* F | / | *Se. minuta* | 2.623 |
| DZ32 | *Se. minuta* M | / | *Se. minuta* | 2.653 |
| DZ33 | *Se. minuta* M | / | *Se. minuta* | 2.573 |
| DZ34 | *Se. schwetzi* F | *Se. schwetzi* | *Se. schwetzi* | 2.417 |
| DZ35 | *Se. schwetzi* F | *Se. schwetzi* | *Se. schwetzi* | 2.430 |
| DZ36 | *Se. schwetzi* F | *Se. schwetzi* | *n/d* |  |
| DZ38 | *Se. schwetzi* M | *Se. schwetzi* | *n/d* |  |
| DZ81^$^ | *Ph. sergenti* M | / | *Ph. alexandri*^#^ | 2.709 |
| DZ82 | *Ph. longicuspis* M | *Ph. longicuspis* | *Ph. longicuspis* | 2.660 |
| DZ83 | *Ph. longicuspis* M | *Ph. longicuspis* | *Ph. longicuspis* | 2.650 |
| DZ84 | *Ph. sergenti* M | / | *Ph. sergenti* | 2.664 |
| DZ85 | *Ph. longicuspis* M | *Ph. longicuspis* | *Ph. longicuspis* | 2.624 |
| DZ86 | *Ph. longicuspis* M | *Ph. longicuspis* | *Ph. longicuspis* | 2.712 |
| DZ87 | *Ph. papatasi* M | / | *Ph. papatasi* | 2.609 |
| DZ88 | *Ph. papatasi* M | / | *Ph. papatasi* | 2.535 |
| DZ89 | *Ph. sergenti* M | / | *Ph. sergenti* | 2.659 |
| DZ90 | *Ph. papatasi* F | / | *Ph. papatasi* | 2.313 |
| DZ91 | *Ph. bergeroti* M | *Ph. bergeroti* | *Ph. bergeroti*^#^ | 2.731 |
| DZ92 | *Ph. papatasi* F | *Ph. papatasi* | *Ph. papatasi* | 2.241 |
| DZ93 | *Ph. papatasi* F | *Ph. papatasi* | *Ph. papatasi* | 2.559 |
| DZ94 | *Ph. papatasi* M | *Ph. papatasi* | *Ph. papatasi* | 2.613 |
| DZ95 | *Ph. longicuspis* F | / | *Ph. longicuspis* | 2.550 |
| DZ96 | *Ph. longicuspis* M | *Ph. longicuspis* | *Ph. longicuspis* | 2.434 |
| DZ97 | *Ph. alexandri* F | / | *Ph. alexandri*^#^ | 2.578 |
| DZ98 | *Ph. alexandri* F | / | *Ph. alexandri*^#^ | 2.612 |
| DZ99 | *Ph. alexandri* M | / | *Ph. alexandri*^#^ | 2.647 |
| DZ100 | *Ph. alexandri* M | / | *Ph. alexandri*^#^ | 2.676 |
| DZ101 | *Ph. bergeroti* F | *Ph. bergeroti* | *Ph. bergeroti*^#^ | 2.788 |
| DZ102 | *Ph. alexandri* M | *Ph. alexandri* | *Ph. alexandri*^#^ | 2.671 |
| DZ103 | *Ph. bergeroti* M | *Ph. bergeroti* | *Ph. bergeroti*^#^ | 2.783 |
| DZ104 | *Ph. papatasi* F | / | *Ph. papatasi* | 2.599 |
| DZ105 | *Ph. papatasi* M | *Ph. papatasi* | *Ph. papatasi* | 2.527 |
| DZ106 | *Ph. alexandri* M | / | *Ph. alexandri*^#^ | 2.752 |
| DZ107 | *Ph. alexandri* M | / | *Ph. alexandri*^#^ | 2.825 |
| DZ108 | *Ph. bergeroti* M | *Ph. bergeroti* | *Ph. bergeroti*^#^ | 2.862 |
| DZ109 | *Ph. papatasi* F | / | *Ph. papatasi* | 2.209 |
| DZ110 | *Ph. papatasi* F | / | *Ph. papatasi* | 2.064 |
| DZ111 | *Ph. bergeroti* F | *Ph. bergeroti* | *Ph. bergeroti*^#^ | 2.725 |
| DZ112 | *Ph. bergeroti* M | *Ph. bergeroti* | *Ph. bergeroti*^#^ | 2.601 |
| DZ113 | *Ph. alexandri* F | / | *Ph. alexandri*^#^ | 2.730 |
| DZ114 | *Ph. papatasi* F | *Ph. papatasi* | *Ph. papatasi* | 2.407 |
| DZ115 | *Ph. papatasi* M | *Ph. papatasi* | *Ph. papatasi* | 2.308 |
| DZ116 | *Ph. papatasi* F | / | *Ph. papatasi* | 2.257 |
| DZ117 | *Ph. papatasi* M | *Ph. papatasi* | *Ph. papatasi* | 2.458 |

M: male; F: female; -: Impossible to sequence; **/**: Not sequenced; n/d: Not determined; ^#^: Used to upgrade in-house reference database; ^$^: Species identity verified by MALDI-TOF MS; LSV: Log score value provided by MALDI BioTyper
