# Supplementary material for: Phlebotomine sand fly survey, blood meal source identification, and description of Sergentomyia imihra n. sp. in the central Sahara of Algeria
Source: Parasit Vectors. 2024 Nov 4;17:449. doi: 10.1186/s13071-024-06542-9 (PMC11536750; doi:10.1186/s13071-024-06542-9)
Supplement: Supplementary file 8 — Additional file 8: Table S6. Result summary of blood meal analysis using PMM-MALDI-TOF mass spectrometry. [file 13071_2024_6542_MOESM8_ESM.docx]

**Additional file 8: Table S6.** Result summary of blood meal analysis using PMM-MALDI-TOF mass spectrometry.

| Species  Blood meal | *Ph. alexandri* | *Ph. papatasi* | *Ph. longicuspis* | *Ph. perniciosus* | Total |
| --- | --- | --- | --- | --- | --- |
| Camel | 2 | 3 | 0 | 0 | 5 |
| Dog | 1 | 4 | 0 | 0 | 5 |
| Donkey | 0 | 0 | 1 | 1 | 2 |
| Goat | 1 | 11 | 0 | 0 | 12 |
| Horse | 1 | 0 | 0 | 0 | 1 |
| Human | 1 | 3 | 0 | 0 | 4 |
| Human + goat | 0 | 1 | 0 | 0 | 1 |
| Chicken | 0 | 1 | 0 | 0 | 1 |
| Sheep | 0 | 2 | 0 | 0 | 2 |
| Total | 6 | 25 | 1 | 1 | 33 |
