# Supplementary material for: Phlebotomine sand fly survey, blood meal source identification, and description of Sergentomyia imihra n. sp. in the central Sahara of Algeria
Source: Parasit Vectors. 2024 Nov 4;17:449. doi: 10.1186/s13071-024-06542-9 (PMC11536750; doi:10.1186/s13071-024-06542-9)
Supplement: Supplementary file 4 — Additional file 4: Table S2. Genetic distances of Cox I sequences of Algerian Sergentomyia species estimated using Tamura 3-parameter model. The number of base substitutions per site from between sequences are shown. The rate variation among sites was modeled with a gamma distribution. [file 13071_2024_6542_MOESM4_ESM.pdf]

[	1	2	3	4	5	6	7	8	9	10	11	12	13	14	15	16	17	18	19	20	21	22	23	24	25	26	27	28	29	30	31	32	33	34
[1]																																		
[2]	0.167																																	
[3]	0.164	0.011																																
[4]	0.161	0.009	0.002																															
[5]	0.161	0.006	0.006	0.004																														
[6]	0.161	0.044	0.041	0.041	0.041																													
[7]	0.161	0.015	0.015	0.013	0.009	0.044																												
[8]	0.170	0.015	0.015	0.013	0.009	0.048	0.019																											
[9]	0.153	0.017	0.013	0.011	0.011	0.037	0.017	0.021																										
[10]	0.164	0.011	0.011	0.009	0.006	0.043	0.015	0.015	0.017																									
[11]	0.170	0.017	0.019	0.017	0.013	0.054	0.023	0.023	0.025	0.011																								
[12]	0.167	0.017	0.019	0.017	0.013	0.045	0.023	0.023	0.025	0.019	0.007																							
[13]	0.161	0.006	0.006	0.004	0.000	0.041	0.009	0.009	0.011	0.006	0.013	0.013																						
[14]	0.164	0.046	0.043	0.044	0.044	0.002	0.046	0.050	0.039	0.046	0.056	0.047	0.044																					
[15]	0.161	0.044	0.041	0.041	0.041	0.000	0.044	0.048	0.037	0.043	0.054	0.045	0.041	0.002																				
[16]	0.167	0.175	0.175	0.172	0.172	0.182	0.163	0.181	0.166	0.175	0.183	0.183	0.172	0.185	0.182																			
[17]	0.161	0.172	0.172	0.169	0.169	0.178	0.166	0.177	0.163	0.172	0.180	0.180	0.169	0.181	0.178	0.009																		
[18]	0.161	0.175	0.174	0.172	0.172	0.181	0.169	0.180	0.166	0.174	0.183	0.183	0.172	0.184	0.181	0.011	0.002																	
[19]	0.164	0.175	0.174	0.172	0.172	0.181	0.169	0.180	0.166	0.174	0.183	0.183	0.172	0.184	0.181	0.009	0.004	0.006																
[20]	0.162	0.172	0.172	0.169	0.169	0.179	0.166	0.178	0.164	0.172	0.180	0.181	0.169	0.182	0.179	0.009	0.000	0.002	0.004															
[21]	0.164	0.175	0.174	0.172	0.172	0.181	0.169	0.180	0.166	0.174	0.183	0.183	0.172	0.184	0.181	0.009	0.004	0.006	0.000	0.004														
[22]	0.187	0.181	0.175	0.178	0.178	0.172	0.172	0.187	0.172	0.187	0.190	0.187	0.178	0.172	0.172	0.186	0.181	0.184	0.184	0.181	0.184													
[23]	0.187	0.187	0.182	0.184	0.184	0.172	0.178	0.193	0.178	0.193	0.193	0.184	0.184	0.172	0.172	0.195	0.190	0.193	0.193	0.190	0.193	0.007												
[24]	0.193	0.187	0.181	0.184	0.184	0.178	0.178	0.193	0.178	0.193	0.193	0.190	0.184	0.178	0.178	0.192	0.187	0.190	0.190	0.187	0.190	0.004	0.007											
[25]	0.166	0.192	0.186	0.186	0.186	0.173	0.186	0.195	0.177	0.189	0.201	0.195	0.186	0.176	0.173	0.163	0.163	0.166	0.157	0.163	0.157	0.181	0.181	0.187										
[26]	0.169	0.202	0.196	0.196	0.196	0.176	0.189	0.205	0.186	0.198	0.211	0.205	0.196	0.179	0.176	0.166	0.166	0.169	0.160	0.166	0.160	0.184	0.184	0.190	0.009									
[27]	0.172	0.205	0.199	0.199	0.199	0.185	0.192	0.208	0.189	0.202	0.214	0.208	0.199	0.188	0.185	0.166	0.166	0.169	0.160	0.166	0.160	0.187	0.187	0.193	0.007	0.009								
[28]	0.166	0.192	0.186	0.186	0.186	0.173	0.186	0.195	0.177	0.189	0.201	0.195	0.186	0.176	0.173	0.163	0.163	0.166	0.157	0.163	0.157	0.181	0.181	0.187	0.000	0.009	0.007							
[29]	0.166	0.199	0.193	0.192	0.192	0.179	0.186	0.202	0.183	0.195	0.208	0.201	0.192	0.182	0.179	0.163	0.163	0.166	0.157	0.163	0.157	0.181	0.181	0.187	0.004	0.009	0.004	0.004						
[30]	0.161	0.050	0.048	0.048	0.048	0.041	0.046	0.054	0.048	0.054	0.060	0.056	0.048	0.043	0.041	0.184	0.181	0.184	0.184	0.181	0.184	0.175	0.181	0.181	0.170	0.173	0.182	0.170	0.176					
[31]	0.161	0.054	0.052	0.052	0.052	0.041	0.050	0.059	0.048	0.059	0.065	0.060	0.052	0.043	0.041	0.187	0.184	0.187	0.187	0.184	0.187	0.175	0.181	0.181	0.173	0.176	0.186	0.173	0.179	0.004				
[32]	0.158	0.052	0.050	0.050	0.050	0.039	0.048	0.056	0.046	0.057	0.063	0.058	0.050	0.041	0.039	0.184	0.181	0.184	0.184	0.181	0.184	0.172	0.178	0.178	0.170	0.173	0.182	0.170	0.176	0.002	0.002			
[33]	0.167	0.059	0.056	0.057	0.057	0.046	0.059	0.063	0.052	0.063	0.069	0.065	0.057	0.048	0.046	0.193	0.190	0.193	0.193	0.190	0.193	0.175	0.181	0.180	0.167	0.170	0.179	0.167	0.173	0.011	0.011	0.009		
[34]	0.176	0.057	0.052	0.050	0.050	0.057	0.052	0.056	0.045	0.056	0.065	0.065	0.050	0.059	0.057	0.193	0.190	0.193	0.193	0.190	0.193	0.193	0.199	0.199	0.177	0.186	0.183	0.177	0.177	0.033	0.033	0.031	0.037	
[35]	0.160	0.196	0.192	0.190	0.190	0.186	0.183	0.199	0.183	0.199	0.197	0.194	0.190	0.189	0.186	0.179	0.182	0.185	0.185	0.182	0.185	0.175	0.178	0.178	0.165	0.172	0.175	0.165	0.169	0.168	0.168	0.165	0.168	0.193

[1] #ON548470 [2] #OQ194091 [3] #OQ194096 [4] #OQ194097 [5] #OQ194098 [6] #OQ194099 [7] #OQ194100 [8] #OQ194105 [9] #OQ194106 [10] #OQ194107
[11] #OQ194108 [12] #OQ194109 [13] #OQ194110 [14] #OQ194112 [15] #OQ194113 [16] #OQ194092 [17] #OQ194101 [18] #OQ194102 [19] #OQ194103 [20] #OQ194104
[21] #OQ194111 [22] #OQ194093 [23] #OQ194094 [24] #OQ194095 [25] #OQ194114 [26] #OQ194115 [27] #OQ194116 [28] #OQ194117 [29] #OQ194118 [30] #MH577087
[31] #MH577094 [32] #MH577069 [33] #MH577068 [34] #MH577067 [35] #OQ194119
